# Mixed methods study of medication-related decision support alerts experienced during electronic prescribing for inpatients at an English hospital

**DOI:** 10.1136/ejhpharm-2017-001483

**Published:** 2018-05-19

**Authors:** Helen Bell, Sara Garfield, Sonia Khosla, Chimnay Patel, Bryony Dean Franklin

**Affiliations:** 1 Pharmacy Department, Charing Cross Hospital, Imperial College Healthcare NHS Trust, London, UK; 2 UCL School of Pharmacy, London, UK

**Keywords:** hospitals, decision support, england, electronic prescribing, alerts

## Abstract

**Objectives:**

Electronic prescribing and medication administration systems are being introduced in many hospitals worldwide, with varying degrees of clinical decision support including pop-up alerts. Previous research suggests that prescribers override a high proportion of alerts, but little research has been carried out in the UK. Our objective was to explore rates of alert overriding in different prescribing situations and prescribers’ perceptions around the use of decision support alerts in a UK hospital.

**Methods:**

We conducted a mixed methods study on three cardiology wards, directly observing medical and non-medical prescribers’ alert override rates during both ward round and non-ward round prescribing; observations were followed by semi-structured interviews with prescribers, which were then transcribed and analysed thematically.

**Results:**

Overall, 69% of 199 observed alerts were overridden. Alerts experienced during ward rounds were significantly more likely to be overridden than those outside of ward rounds (80% of 56 vs 51% of 63; p=0.001, Χ^2^ test). While respondents acknowledged that alerts could be useful, several also described negative unintended consequences. Many were of the view that usefulness of alerts was limited if the alert was reminding them to do something they would do anyway, or suggesting something they did not feel was relevant. Findings suggest that targeting, timing and additional features of alerts are critical factors in determining whether they are acted on or overridden.

**Conclusion:**

The majority of alerts were overridden. Alerts may be less likely to be overridden if they are built into the prescribing workflow.

## Introduction

Electronic prescribing and medication administration (ePMA) systems are increasingly prevalent in the inpatient setting. ePMA systems generally incorporate clinical decision support (CDS) aimed at aiding prescribers in prescribing safely, such as drug dictionaries, default dose suggestions, drug-drug interaction alerts, drug-allergy alerts and alerts to relevant laboratory results.^1^ Pop-up alerts are often used to highlight warnings to the prescriber.^1^

There is some evidence that ePMA incorporating CDS alerts reduces medication errors^2–6^ and improves practitioner performance^7–9^ in both inpatient and outpatient settings. However, alert fatigue limits its use. The risk of ignoring or overriding important alerts therefore rises with an increase in the number of less relevant alerts due to users being overwhelmed by, and desensitised to, alert presentation.^10 11^ A systematic review in hospital and primary care settings found that 49%–96% of alerts were overridden or ignored by prescribers and emphasised the importance of their timing and frequency.^11^ Another review identified that the most important reasons for overriding an alert were the alert not being perceived as serious or relevant, or being shown repeatedly.^12^ Alerts have also been found to have been inappropriately targeted, with too much information already known to prescribers, and requiring redesign.^9^ In addition, alerts may have a negative impact on prescribers’ knowledge^13^ and concerns have been raised about inexperienced doctors becoming too dependent on CDS.^14^

The majority of the research in this field has been conducted outside the UK, and may not be generalisable to the UK due to differences in the way medicines are prescribed, dispensed and administered.^15 16^ To our knowledge, no studies have been carried out in a UK adult setting. We therefore conducted an exploratory study of prescribers’ experiences of CDS alerts in an English hospital. Our objectives were to determine the proportion of alerts that were overridden in different prescribing situations, and to explore prescribers’ experiences and perceptions around use of CDS alerts in the inpatient setting.

## Methods

### Setting

The study took place in a 349 bed London teaching hospital during November and December 2016. Inpatient ePMA had been introduced a year previously as part of a commercially available electronic patient record system. There were two types of pop-up alert in use. These appeared either when the patient details were accessed (type 1) or when the medication page was opened (type 2). Type 1 alerts, such as the antibiotic review alert, continued to be displayed until they had been acted on. To access the patients’ details the user could acknowledge the alert, which meant it would then appear again next time the patient’s records were accessed. Type 2 alerts required action before medication could be prescribed. For example, the venous thromboembolism (VTE) risk assessment alert could be closed either by completing the VTE assessment or by clicking an override button and giving an override reason.

At the time of the study, one type 1 and five type 2 alerts were in use. The study took place on three cardiac wards: cardiothoracic (ward A; 24 beds), cardiology (ward B; 24 beds) and cardiothoracic critical care (ward C; 16 beds). On ward A, advanced nurse practitioner independent prescribers did a considerable proportion of prescribing, whereas only medical staff prescribed on the other two wards. During ward rounds, senior doctors generally made the prescribing decisions and more junior doctors or nurse prescribers carried out the prescribing tasks.

### Study design

This was a descriptive mixed methods study, comprising quantitative observations of ward round and non-ward round prescribing, followed by semi-structured qualitative interviews with medical and non–medical prescribers. The study was registered as a service evaluation within the hospital; no personally identifiable data were recorded for patients or healthcare professionals. Two final-year pharmacy students were trained to collect the data.

### Data collection

#### Quantitative observations

Following approval from ward managers and lead consultants, individual prescribers on the study wards were given information leaflets and invited to give written consent to be observed. Observations took place every day for five consecutive weekdays on each ward, starting during the morning ward round (about 08:00 hours) and continuing until mid-afternoon (about 15:00 hours), to enable observation of both ward round and non-ward round prescribing. During non-ward round prescribing they observed prescribing in the doctors’ office, as this was where most prescribing took place. The students collected data on different wards simultaneously.

We documented the frequency and types of alerts related to medication experienced, by whom, the observed actions taken and any further contextual information (see online supplementary appendix). If the same prescriber was observed experiencing the same alert more than once for the same patient interaction, the observed alert was documented only once. However, if a different prescriber was observed viewing the same alert for the same patient, or if the original prescriber returned to a patient’s record later and the same alert was triggered, then these were documented as new alerts.

10.1136/ejhpharm-2017-001483.supp1 Supplementary file 1

#### Semi-structured interviews

Following the observations, the healthcare professionals observed on the ward rounds were invited to give informed written consent to take part in an interview. During the interviews, participants were asked to provide their opinions on the value of alerts during prescribing and on the positive and negative features of alerts. Respondents were also asked about which alerts were useful, their views on timing of alerts and the effect of alerts on patient safety. Prescribers who had been observed experiencing alerts were asked about their experience of these and about why they had taken the specific actions observed.

### Analysis

Data on the frequency and types of alert were presented descriptively. Our dependent variable was action taken in response to CDS alert. Our independent variables were alert timing (ward round vs non-ward round prescribing), alert type (the antibiotic review alert vs VTE risk assessment alert) and prescriber type (medical vs non-medical prescribers). We used Χ^2^ test or Fisher’s exact test^17^ depending on the sample size. We then carried out a logistic regression analysis, including all independent variables that were found to be significant at the p=0.01 level in the bivariate analyses.

Interviews were audio-recorded, anonyomised and transcribed verbatim. Inductive thematic analysis was then conducted without any pre-existing coding framework. The students reviewed the transcripts to identify recurrent themes. The interview transcripts were considered in context with observational data where prescribers had also been observed using the ePMa system. Two students analysed half of the interview data each and a researcher then checked and merged the analysis and coding.

## Results

### Observations

During the 15-day study period, we observed 41 hours 24 min of ward rounds and 53 hours 40 min of non-ward round activities. Eighteen healthcare professionals were observed (table 1).

**Table 1:** Type and number of healthcare professionals observed during both ward round and non-ward round prescribing

Ward	Numbers of each type of prescriber observed				Total
	Medical			Non-medical	
	Foundation doctors	Registrars	Consultants	Nurse prescribers	
A	1	–	–	2	3
B	3	4	2*	–	9
C	6	–	–	–	6
Total	10	4	2	2	18

*Consultants on ward B led the ward round, but did not prescribe using the electronic and medicine administration system (ePMA)—prescribing decisions were transcribed to ePMA by junior doctors during the ward round.

A total of 119 CDS alerts related to medication were observed, all of which related to either antibiotic review (n=85) or VTE (n=34). Fifty-six (47%) were observed during the ward round and 63 (53%) during non-ward round prescribing.

Based on univariate analysis, significantly more VTE alerts (p=0.0003) were acted on during non-ward round prescribing than ward round prescribing (table 2). However, there was no significant difference (p=0.11) in relation to the antibiotic review alert. Overall, there was no significant difference between VTE alerts and antibiotic review alerts in the action taken (p=0.505). However, overall, alerts were significantly more likely to be overridden during ward round prescribing (p=0.001) as opposed to non-ward round prescribing. Medical prescribers were significantly more likely to override alerts (p<0.001). No alerts were overridden by non-medical prescribers.

**Table 2:** Observed actions in relation to alert type presented to prescriber during ward round and non-ward round prescribing

	Antibiotic review alert		Venous thromboembolism alert	
	Acted on alert	Alert overridden	Acted on alert	Alert overridden
Ward round observations	10 (24%)*	31 (76%)	1† (7%)	14 (93%)
Non-ward round observations	18 (41%)*	26 (59%)	13 (68%)†	6 (32%)
Total number of alerts	85		34	

*There was no significant difference (p=0.11) between ward and non-ward round prescribing (p=0.1).

†Significantly more alerts (p=0.0003) were acted on during non-ward round prescribing than ward round prescribing.

Ward round versus non-ward round prescribing remained a significant variable (p=0.03) in logistic regression analysis but the difference between medical and non-medical prescribers was no longer significant due to the confounding effect of ward round versus non-ward round prescribing.

### Semi-structured interviews

Sixteen interviews were conducted, 14 with medical prescribers and 2 with non-medical prescribers. The duration of interviews ranged from 4 to 34 min. Perceived usefulness of alerts

Interviewees were generally of the view that alerts could be useful and act as reminders. In particular, although not observed during our study, the allergy alert was considered helpful and important by all but one respondent who discussed this alert. The remaining respondent was of the view that it was not necessary but gave no reason.

That’s always useful. That’s probably one of the most important alerts. Because when you see so many patients, in your head you know that someone has got an allergy, but what if you are doing something quickly there. (Interview 7)

Two respondents also made reference to VTE assessments being important to the hospital organisation, as they received payment for their completion as part of a Commissioning for Quality and Innovations incentive target. Another respondent expressed the view that alerts could increase junior doctors’ confidence in prescribing.

However, many were also of the view that usefulness of alerts could be limited if the alert was reminding them to do something they would do anyway (antibiotic review) or asking them to do something they did not feel was relevant (VTE assessment). Some respondents were of the view that medication was reviewed during the ward round already. This was particularly the case on surgical wards where it was perceived that VTE prophylaxis would always be prescribed.

It makes you realize the VTE (venous thromboembolism prophylaxis) is not being prescribed for that patient but again for our setting it’s not really … relevant for us post-surgery. (Interview 16)

Respondents were also of the view that alerts were only as useful as the accuracy of the information on which they were based. They were therefore dependent on fields having being correctly filled in. For example, the allergy alert would only be helpful if the allergies had been documented and the medication review alert would only be useful if the correct review date had been specified when the antibiotic was first prescribed. Some interviewees perceived that default practice was to put the prescribing date as the review date, which led to the alert popping up incorrectly and being overridden.

If the person prescribing the drugs has put an inappropriate review date, then it is just really annoying. (Interview 10)

#### Factors affecting action taken to alerts

Interviewees suggested that the timing of alerts and where they were placed in relation to workflow was a critical factor in determining whether they would be acted on or overridden. The allergy alert appeared when prescribers tried to prescribe a medication that the patient was allergic to, at the point of prescribing; this was considered helpful. Antibiotic review alerts appeared when a patient’s record was opened. These therefore appeared during the ward round which was considered helpful as this was when medication was typically reviewed. However, some respondents were of the opinion that it would be even better placed at the point of opening the patient’s medication prescribing screen as it would then appear at the time medicines were specifically being discussed. The VTE assessment was considered by respondents to be in an incorrect place in the workflow as it appeared when medicines were being prescribed. Medicines were typically prescribed by junior doctors during the ward round and they had insufficient time to carry out the VTE assessment before the ward round moved to the next patient. The assessments would be completed as a task in the afternoon and respondents suggested that it was at this point that a reminder may be more helpful.

Unless you are doing the assessment on the ward round, which no one does, because we are trying to get drugs written and trying to do loads of different things. What it ultimately is… it’s almost like it’s set up to not be successful… I just think the timing is probably not appropriate. (Interview 15)

In addition to the timing of alerts, it became apparent from the analysis that they also needed to be targeted to the right person, for example, junior versus senior staff, and the patient’s regular clinical team versus on-call staff who may not know the patient as well.

That was the first time I was meeting that patient and I didn’t know enough of the background to be able to fill out the VTE (venous thromboembolism) prophylaxis form accurately. (Interview 11)

Interview data also suggested that alerts were more likely to be acted on if they were user-friendly. Specific improvements suggested including links from the antibiotic review alert to the antibiotic prescription concerned, and making the VTE form more accessible as a quick link as part of the alert.

#### Effect of alerts on patient safety

Overall, views on the effects of alerts on patient safety were mixed. While it was acknowledged that alerts could bring important safety-related information to the attention of prescribers, several respondents described negative unintentional consequences of the alerts. These included loss of concentration leading to medication errors, delays in prescribing urgent medication and intended medication not being prescribed as a result of the override button not being pressed. These negative consequences were described frequently in relation to the VTE alert as this popped up for every medicine prescribed and the medicine could not be prescribed until the VTE assessment was completed or the override button was pressed and a reason given.

You can sometimes get to the point where you are clicking without sometimes looking, if you have just prescribed a whole stack of drugs, er, and you are trying to get them onto the system, so I have on occasion forgotten to switch the radio (override) button and someone wandered up to me and said why is their potassium replacement not prescribed and then I think oh I’ve prescribed everything but not this one because I’ve forgotten to switch the radio (override) button to emergency override. (Interview 13)

## Discussion

### Key findings

Triangulation of quantitative and qualitative data suggests that putting alerts in the correct place in the prescriber’s workflow would facilitate their being acted on. VTE assessments were significantly less likely to be acted on if they appeared during a ward round than during non-ward round prescribing, whereas there was no significant difference between the number of antibiotic review alerts acted on during ward round and non-ward round prescribing. Interview data suggest that the reason for this difference was that medication was reviewed as part of the ward round but that VTE assessments were generally carried out in the afternoon and could not easily be incorporated into the ward round.

### Comparison with existing literature

The override rate (69%) observed in this study is consistent with previous research that has found override rates between 49% and 96%.^11 18–20^ The findings demonstrate that the need to target alerts at the correct time and frequency, to the correct prescriber that have been identified in other countries, for example, the USA^9 11 12^ is also critical in the UK. Baysari *et al* 9 noted that non-ward round timing may be preferable for alerts, a finding consistent with our study. In most instances, ward rounds are led by senior doctors with one or more junior doctor in attendance. Both Baysari *et al* 9 and the present study found that senior doctors making prescribing decision during ward rounds are often unaware of any CDS alerts that are triggered by those prescribing decisions as they do not enter the medication order into the ePMA system. This role is taken on by the most junior members of staff in time-pressured situations. This time pressure may explain why antibiotic review dates are often given the default of the same date as the initial prescription. The importance of the medication information in the ePMA system being up-to-date to avoid false alerts is supported by previous work.^21^

Van der Sijs *et al* 11 suggested that requiring entry of reasons for overriding alerts triggers physicians to rethink the potential unsafe situation gives more insight to other healthcare professionals and can help adjust the knowledge database. Scott *et al* 13 found prescribers who were shown alerts that did not require action were 3.6 times more likely to make a prescribing error than those shown alerts that required action. However, our study suggests that requiring reasons for overrides (type 2 alerts) may lead to increased negative consequences as it may reduce concentration and lead to important tasks not being completed due to interruption. Slight *et al* 22 also found that reasons given for overrides were not always appropriate.

### Strengths and weaknesses

Strengths of the study are the mixed methods approach, inclusion of observation of both ward round and non-ward round prescribing and the inclusion of non-medical prescribers, allowing comparisons to be made. The main limitations are that only one specialty area was included in one hospital using one ePMA system and we did not include weekends or explore other variables such as alert wording or positioning, nor the clinical appropriateness of the overrides observed. Prescribers may have behaved in a different way as a result of being observed, and we only observed the antibiotic review alert and VTE alert. We did not measure inter-rater reliability between observers. Our sample size was fairly small (n=119 alerts).

### Implications for practice and research

This study has identified areas for hospitals and system vendors to address in order to optimise the way users experience and respond to CDS alerts. Specifically, our findings suggest that alerts should be targeted to the right person at the right time with electronic links to the action required where relevant. Prescribers and other users should be involved in design of prescribing workflows and the use of CDS alerts.

## Conclusion

In a small study in an English hospital we found that the majority of decision support alerts were overridden, but VTE alerts were significantly less likely to be overridden when they appeared outside of ward rounds. The findings suggest that alerts are less likely to be overridden if they are built into the correct workflows of prescribers both in terms of the timing of the alert and the healthcare professionals targeted. Findings also suggest that alerts should include links to the action needed.

**What this paper adds**

**What is already known on this subject**

- Research from other countries suggests that prescribers override a high proportion of clinical decision support alerts.
- Little research has been carried out in the UK.

**What this study adds**

- Override rates in the UK are similar to the other countries.
- The timing of alert pop ups is independently and significantly associated with whether alerts are acted on or overridden.
