# Supplementary material for: Mixed methods study of medication-related decision support alerts experienced during electronic prescribing for inpatients at an English hospital
Source: Eur J Hosp Pharm. 2018 May 19;26(6):318–22. doi: 10.1136/ejhpharm-2017-001483 (PMC6855857; doi:10.1136/ejhpharm-2017-001483)
Supplement: Supplementary file 1 [file ejhpharm-2017-001483supp001.pdf]

Appendix:  Data collection form

Ward Round Duration: ______________________ Number of patients seen: ____________________________

| Ward: | | | | Date: | | No. of COWs |
|---|---|---|---|---|---|---|
| Alert Number | Bed No. & Patient initials | Type of Alert | Alert seen in which screen | Action taken | Who/Name | Comments / |
| 1 | | | | | | |
| 2 | | | | | | |
| 3 | | | | | | |
| 4 | | | | | | |
| 5 | | | | | | |
| 6 | | | | | | |
| 7 | | | | | | |
| 8 | | | | | | |
| 9 | | | | | | |

<u>Types of alerts</u>

Medication review = MR
Allergy Alert = AA
Allergy not documented = AND
Venous thromboembolism = VTE
High Alert = HA
Non-Formulary Alert = NF
Other = O

<u>Alert seen in which screen</u>

Front Page = FP
Prescribing Page = PP
Other = O

<u>Who</u>

Consultant = C
Registrar = R
Foundation Year = FY
Non-Medical = NM
